# How similar is “similar,” or what is the best measure of soil spectral and physiochemical similarity?

**DOI:** 10.1371/journal.pone.0247028

**Published:** 2021-03-25

**Authors:** R. Zeng, J. P. Zhang, K. Cai, W. C. Gao, W. J. Pan, C. Y. Jiang, P. Y. Zhang, B. W. Wu, C. H. Wang, X. Y. Jin, D. C. Li

**Affiliations:** 1 School of Geography Science, Nanjing University of Information Science and Technology, Nanjing, China; 2 Upland Flue-cured Tobacco Quality & Ecology Key Laboratory of China Tobacco Guizhou Academy of Tobacco Science, Guiyang, China; 3 College of Resources and Environment, Southwest University, Chongqing, China; 4 China National Tobacco Corporation Guizhou Provincial Company, Guiyang, China; 5 State Key Laboratory of Soil and Sustainable Agriculture, Institute of Soil Science, Chinese Academy of Sciences, Nanjing, China; ICAR-Central Arid Zone Research Institute, INDIA

## Abstract

Spectral similarity indices were used to select similar soil samples from a spectral library and improve the predictive accuracy of target samples. There are many similarity indices available, and precisely how to select the optimum index has become a critical question. Five similarity indices were evaluated: Spectral angle mapper (SAM), Euclidean distance (ED), Mahalanobis distance (MD), SAM_pca and ED_pca in the space of principal components applied to a global soil spectral library. The accordance between spectral and compositional similarity was used to select the optimum index. Then the optimum index was evaluated if it can maintain the greatest predictive accuracy when selecting similar samples from a spectral library for the prediction of a target sample using a partial least squares regression (PLSR) model. The evaluated physiochemical properties were: soil organic carbon, pH, cation exchange capacity (CEC), clay, silt, and sand content. SAM and SAM_pca selected samples were closer in composition compared to the target samples. Based on similar samples selected using these two indices, PLSR models achieved the highest predictive accuracy for all soil properties, save for CEC. This validates the hypothesis that the accordance information between spectral and compositional similarity can help select the appropriate similarity index when selecting similar samples from a spectral library for prediction.

## 1. Introduction

Visible and near-infrared (VNIR) spectroscopy has demonstrated its ability to predict many soil physiochemical properties, such as soil organic matter (SOM), particle size, and iron content [1–3]. In addition to its wide use for soil properties, comparison of spectra from soil samples is used in several soil science-related applications [4], such as forensic soil science, archeology, and soil pollution assessments. Similarity indices have also been used to select samples from spectral libraries [5, 6], and build local models for improving the physiochemical prediction of the target site [7–9]. The hypothesis of this strategy is that the selected similar spectra can better represent the spectral features of the target samples, thus leading to better model performance [10, 11].

There are several spectral similarity indices, each with its own quantification, and different indices present different results of sample similarity [10]. For example, the spectral angle mapper (SAM; [12]) measures the angle between two spectral vectors to quantify similarity, while Euclidean distance (ED) measures distance in two or three dimensional Euclidean space. How to select similar spectra is a critical question, as it determines which candidate samples will be included for subsequent model building. Calibration datasets based on different indices will be unique, and thus lead to different model performances. In addition to the question of which spectral similarity index is most suitable for model calibration, the extent to which the index represents other measures of similarity between the soils was also explored; i.e., do similar spectra correspond to similar physiochemical properties of these soils? We may hope so, since the calibrated spectral library will be used to infer the physiochemical properties of the target samples.

Most previous research has examined one familiar or widely applied similarity index [8], with no attempt to compare between indices. Ramirez-Lopez et al. [13] was a notable exception, and developed an indicator to select the optimum similarity index. They proposed that the best distance metric would more accurately reflect soil compositional similarity, and imply (but do not confirm) that this would lead to the best predictive performance of the calibrated model. This was tested by comparing the spectral and compositional similarities of two soil properties: clay and pH. Clay has a strong spectral response in the VNIR around the water absorption features [14], while pH does not. Their research, however, did not cover soil properties with remarkably strong spectral responses, such as SOM [2].

We believe it is necessary to evaluate this method with more physiochemical properties, considering both properties with direct and indirect spectral responses. Direct spectral responses indicate direct interaction between the soil constituents and the electromagnetic radiation, while indirect responses are primarily based on a correlation with a combination of other soil properties. Moreover, a further step was taken beyond the research of Ramirez-Lopez et al. [13] by evaluating whether the calibration datasets selected by the better similarity index achieved higher predictive accuracy for a target sample.

Therefore, the objectives of this research were: (1) to evaluate five similarity indices using the accordance between spectral and compositional similarity (SOM, pH, CEC, clay, silt and sand) to select an optimum similarity index; and, (2) to determine if the optimum index maintains the greatest predictive accuracy when selecting similar samples from a spectral library for the prediction of a target sample. Our research hypothesis was that the samples selected based on the optimal similarity index would achieve higher predictive accuracy for the target samples.

## 2. Materials and methods

### 2.1 Datasets

This study was carried out using a global soil spectral library, including 785 profiles (3831 generic horizons) selected from the International Soil Reference and Information Center (ISRIC). This library contains VNIR spectra (350–2500 nm, sampling interval 1 nm), geographic location, physical and chemical properties, and soil classification information [15]. These profiles were collected from 58 countries in Africa, Asia, Europe, North America, and South America. Spectral measurements were recorded with a FieldSpec FR spectrometer (Analytical Spectral Devices, Boulder, CO). The data providers reduced the spectra to 216 bands by averaging every 10 bands. Physiochemical properties measured by conventional laboratory methods included soil organic carbon (SOC), sand, silt, clay, pH, and cation exchange capacity (CEC).

We performed several quality control checks, and removed all samples with the sum of particle-size separates > 106% or < 94%, leaving 3,813 samples for further analysis [16]. From these, 500 samples with diverse spectral variations were selected as the test dataset using the Kennard-Stone (KS) algorithm [17], and the remaining 3,313 were used as the training set. Selection was based on the Euclidean distance of spectra as represented by principal components (PC). Before the implementation of the KS algorithm and subsequent distance calculation, reflectance spectra were transformed to absorbance, and baseline effects were corrected by a first-derivative transformation with Savitzky–Golay smoothing [18].

### 2.2 Flowchart

**Fig 1:**
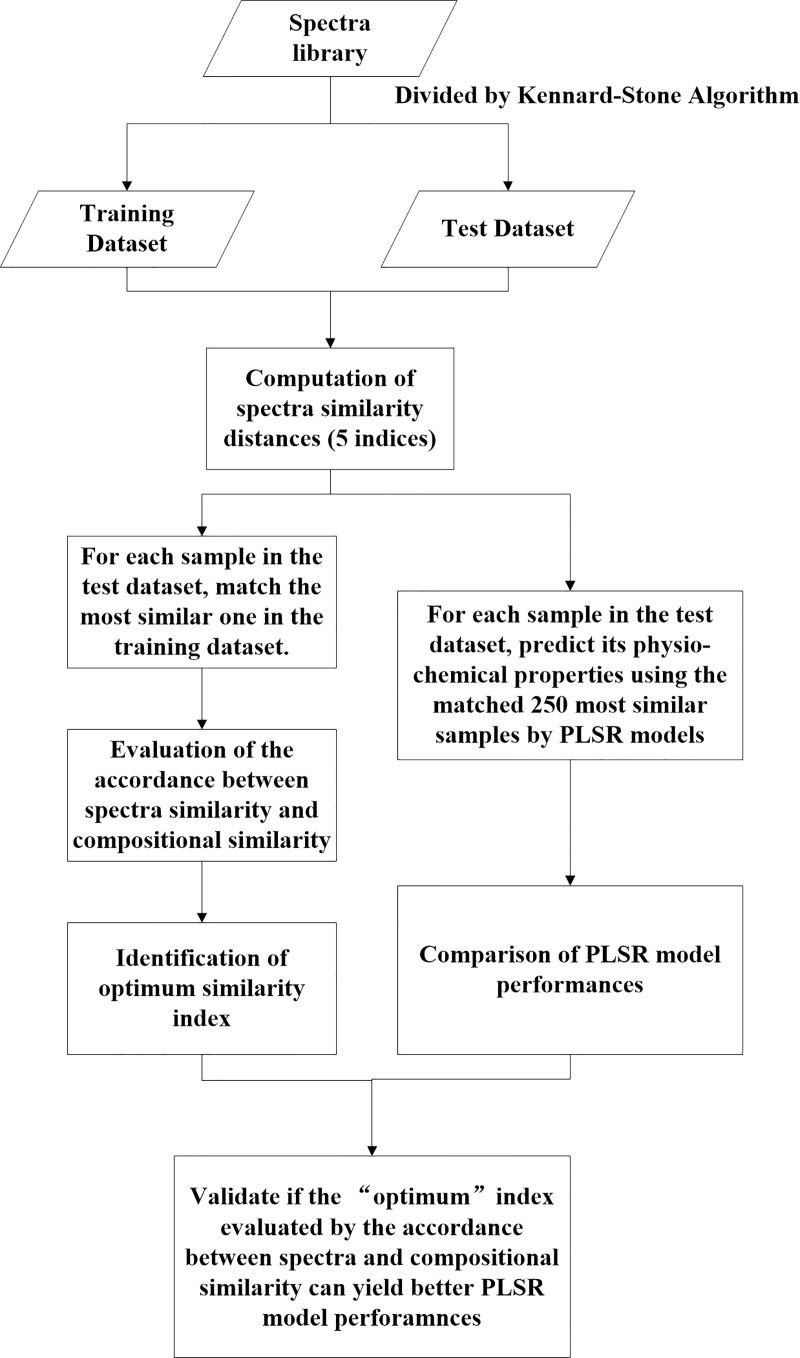
Flowchart of the methodology.

Fig 1 presents the overall procedures of this study, which are explained in further detail below.

### 2.3 Similarity indices

We tested several of the most promising similarity indices presented by Ramirez-Lopez et al. [13] on the pre-processed full spectra, SAM, ED, and MD (see below for a detailed description). In order to remove the effects of collinearity in the predictors (i.e., the spectral bands), we also evaluated SAM (SAM_pca) and ED (ED_pca) in the space of PC. PC analysis was performed in R (*v*. 3.5.2) using the prcomp function of the stats package. We tested different numbers of PCs (1–20), and the similarity distance remained unchanged > 15 PCs, so only the original vectors transformed to 15 PC scores were retained for subsequent analysis.

#### 2.3.1 Spectral Angle Mapper (SAM)

SAM is a commonly used similarity index first introduced by Kruse et al. [12], and measures the spectral angle between different samples. SAM considers both the differences in spectral shape and amplitude (Eq 1):

$$SAM=\cos^{-1}\frac{\sum_{i=1}^{n}U_{i}R_{i}}{{{\left(\sum_{i=1}^{n}{\left(U_{i}\right)}^{2}\right)}^{1/2}(\sum_{i=1}^{n}{{(R}_{i})}^{2})}^{1/2}},$$ (Eq 1)

where *U*_*i*_ and *R*_*i*_ represent the processed spectra for wavelength *i* (or their PC transformation) for the samples in the test and training datasets, respectively; and *n* is the number of spectral bands.

#### 2.3.2 Euclidean Distance (ED)

ED measures the distance of the two spectral vectors in Euclidean space (Eq 2):

$$ED=\sqrt{\sum_{i=1}^{n}{\left(U_{i}-R_{i}\right)}^{2}},$$ (Eq 2)

where *U* and *R* represent the processed spectral vectors (or their PC transformation) for the test and training datasets, respectively; and *n* is the number of spectral bands. ED was calculated using the dist function in the stats package of R.

#### 2.3.3 Mahalanobis Distance (MD)

MD is the distance between two vectors, considering the covariance among vector elements (Eq 3):

$$MD=\sqrt{{\left(U_{i}-R_{j}\right)}^{T}S^{-1}(U_{i}-R_{j})},$$ (Eq 3)

where *U*_*i*_ and *R*_*j*_ are the processed spectral vectors (or their PC transformation) for sample *i* in the test dataset and sample *j* in the training dataset, respectively; and *S* is the covariance matrix between *U*_*i*_ and *R*_*j*_.

### 2.4 Accordance between spectral and individual compositional similarity

The most similar spectra for each of the test set were selected based on the lowest similarity distance for the five indices matched from the training dataset. The six physiochemical properties (pH, SOC, CEC, sand, silt, and clay) of the target samples were then compared to their corresponding matched sample. The root mean square error (RMSE) and the coefficient of determination (R^2^) evaluated from the 1:1 line (actual: predicted) were used to evaluate the accordance between spectral and individual compositional similarity (Eqs 4 and 5):

$$RMSE=\sqrt{\frac{1}{m}\sum_{i=1}^{m}{\left(y_{i}-{\hat{y}}_{i}\right)}^{2}},\;\mathrm{and}$$ (Eq 4)

$$R^{2}=1-\frac{\sum_{i=1}^{m}{\left(y_{i}-{\hat{y}}_{i}\right)}^{2}}{\sum_{i=1}^{m}{\left({\hat{y}}_{i}-\bar{y_{i}}\right)}^{2}},$$ (Eq 5)

where *m* is the number of samples in the test dataset, *y_i_* is the physiochemical property of sample *i* in the test dataset, ${\hat{y}}_{i}$ is the physiochemical property of the most similar sample in the training dataset, and $\bar{y_{i}}$ is the average property value of the matched samples in the training dataset. Higher R^2^ and lower RMSE indicate a greater accordance between the spectra and compositional similarity, and these criteria were used to select the optimum similarity index.

### 2.5 Accordance between spectral and integral compositional similarity

Soil spectra are an integrated result of the physiochemical properties of the sample. Apart from comparing spectra with individual compositional similarity, the relationship between spectral and integral compositional similarity was also investigated. To represent the integral composition, we used six standardized PCs converted from the six physiochemical properties. Since these properties have different units and are correlated, they were scaled first by normal-score transformation, and then converted into six PC scores using the prcomp function of the stats package in R. Then, the PC distance between the target and the matched sample in Euclidean space was calculated to represent the integral compositional similarity (Eq 6):

$$D_{i}=\sqrt{\sum_{i=1}^{6}{\left(U_{pc(i)}-R_{pc(i)}\right)}^{2}},$$ (Eq 6)

where *D*_*i*_ is the integral compositional distance between sample *U* in the test dataset and the most similar sample *R* in the training dataset, as represented by PC; and *U*_*pc(i)*_ and *R*_*pc(i)*_ are the PC scores for the *i*th samples *U* and *R*, respectively, as converted from their corresponding six physiochemical properties.

### 2.6 Partial Least Squares Regression (PLSR) model comparison

The reported optimum similarity indices selected by the accordance between spectral and compositional similarity were evaluated in terms of their predictive power. For each sample in the test dataset, all six properties were predicted by PLSR models based on similar samples matched in the training dataset using the five similarity indices.

The number of similar samples selected from the training dataset has a great effect on the model performance [19], which although important, was not the focus of the research here. Different sizes (n = 5, 10, 20, 30, 40, 50, 100, 150, 200, 250, 300, 400 and 500) were tested for prediction of SOC. Model performance achieved the highest predictive accuracy and stabilized around ~ 250; thus, this size was selected for all subsequent analyses of other physiochemical properties (S1 Fig). PLSR model performance was evaluated by the ratio of percent deviation (RPD; Eq 7):

$$RPD=\frac{SD}{RMSEP},$$ (Eq 7)

where *SD* is the standard deviation of the observed property values for the test dataset, and RMSEP is the RMSE of the prediction (see Eq 4). For each sample in the test dataset, the most similar 250 samples in spectral space (as evaluated by different similarity indices) were selected from the training dataset to build the PLSR models for prediction of the soil properties. We followed the criteria proposed by Chang and Laird [20] to evaluate the performance of the PLSR models: (1) RPD < 1.4, the model is not able to predict the target property; (2) 1.4 ≤ RPD < 2.0, moderate model predictive performance; and (3) 2.0 ≤ RPD < 2.5, the model can predict the target property well.

## 3. Results and discussion

### 3.1 Training and test dataset summary

Table 1 shows the summary statistics of physiochemical properties for the training and test datasets. Since a global soil spectral library was used, the soil properties covered a wide range of values (Table 1). Extremely acidic (pH = 3.00), alkaline (pH = 10.5), sandy with no SOC, and organic soils with high SOC (15.75%) were all included. The particle size class ranged from extremely sandy to extremely clayey. The range of the test set was somewhat narrower as an artifact of the smaller sample size.

**Table 1:** Descriptive statistics of soil physiochemical properties in the training and test datasets.

Dataset	Property	Min	Q1	Q2	Q3	Max	Mean	SD	CV%
Test dataset	pH	3.20	4.80	5.40	6.90	10.50	5.93	1.48	25
	SOC (%)	0.00	0.15	0.37	0.93	15.75	0.95	1.71	181
	CEC (cmol·kg^-1^)	0.00	3.50	8.60	19.83	121.70	14.83	16.70	113
	Sand (%)	0.00	18.38	43.35	71.28	99.20	45.63	30.31	66
	Silt (%)	0.60	11.18	21.00	34.93	84.80	24.81	17.55	71
	Clay (%)	0.00	9.13	25.55	47.03	96.40	29.57	23.14	78
Training dataset	pH	3.00	5.10	6.00	7.40	10.50	6.26	1.42	23
	SOC (%)	0.00	0.23	0.51	1.20	45.80	1.10	1.91	174
	CEC (cmol·kg^-1^)	0.00	5.60	12.10	22.70	189.60	17.08	16.57	97
	Sand (%)	0.00	11.00	31.00	59.20	99.50	37.03	28.32	76
	Silt (%)	0.20	12.90	25.30	43.10	90.50	29.57	20.04	68
	Clay (%)	0.00	16.10	30.90	47.50	96.80	33.39	21.80	65

Q1, Q2, and Q3 are the first, second, and third quartiles, respectively; SD is the standard deviation; and CV% is the percent coefficient of variation.

**Fig 2:**
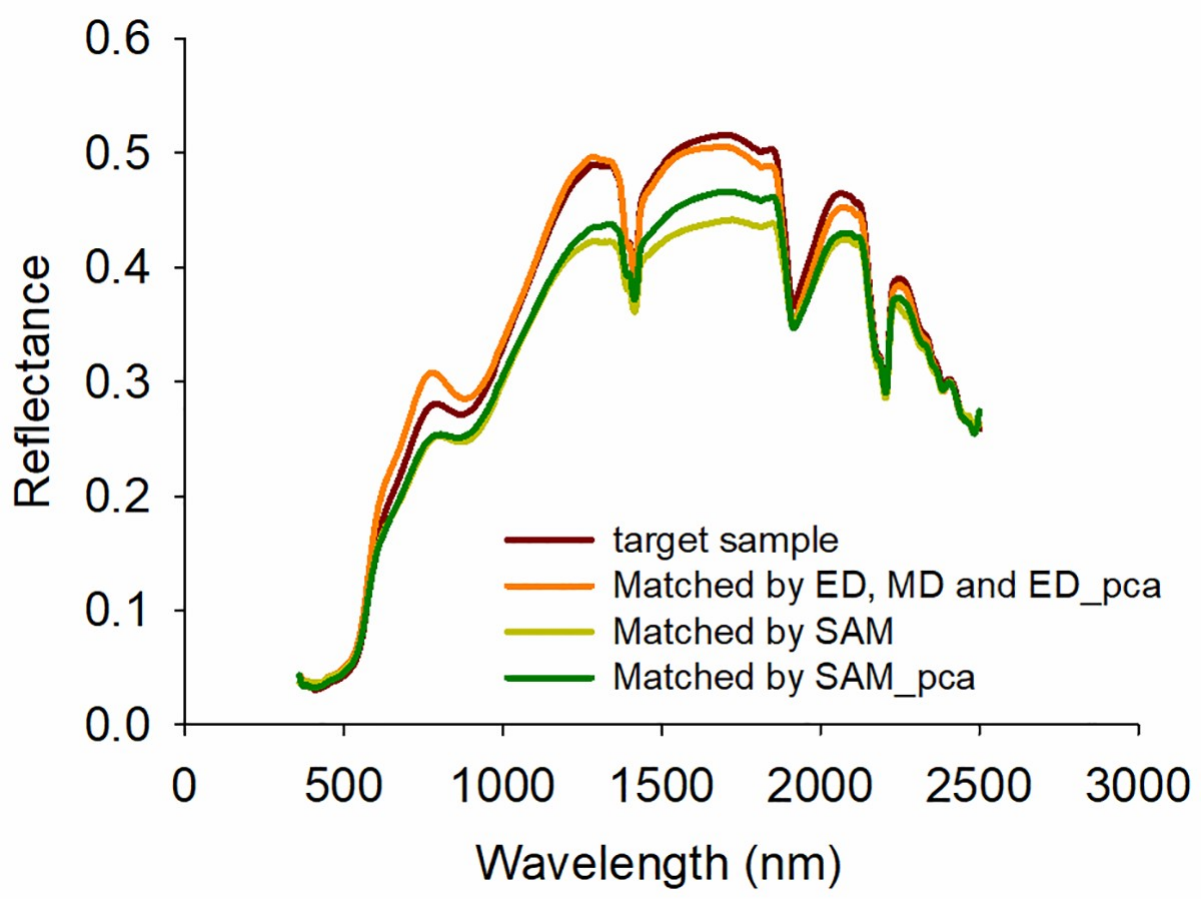
An illustrative example of the most similar spectra selected by the five similarity indices.

Fig 2 presents an illustrative example to depict how the five similarity indices differ in their selection of the most similar spectra. ED, MD, and ED_pca selected the same most similar spectra, primarily because of the similar distance calculations of ED and MD. Their matched spectra nearly overlapped with the target samples because the calculations of ED and MD focus on the relative difference of the reflectance values. The reflectance of the most similar spectra selected by SAM was much lower than that of the target sample since SAM primarily considers similarity in spectral shape. SAM_pca also yielded different results from the other methods.

### 3.2 Comparison between spectral and individual compositional similarity

Table 2 presents a comparison between the spectra and the six individual compositional similarities. The similarity index was selected according to lower RMSE and higher R^2^ values. As these two measurements always agreed, only R^2^ will be presented in the following discussion of the individual soil properties. We used the following criteria [20] to indicate the accordance between spectra and individual compositional similarity: (1) R^2^ < 0.5, poor; (2) 0.5 ≤ R^2^ < 0.8, moderate; and, (3) R^2^ ≥ 0.8, good accordance.

**Table 2:** Accordance of the six physiochemical properties between the samples in the test dataset and their most similar samples matched in the training dataset, by different similarity indices.

Similarity Index	Similarity Indicator	pH	SOC (%)	CEC (cmol·kg^-1^)	Clay (%)	Silt (%)	Sand (%)
SAM	RMSE	0.89	1.15	10.53	14.43	13.95	20.33
	R^2^	0.64	0.55	0.60	0.61	0.37	0.55
ED	RMSE	0.96	1.13	10.52	15.54	15.25	22.92
	R^2^	0.58	0.56	0.60	0.55	0.25	0.43
MD	RMSE	0.93	1.20	10.83	15.48	15.28	22.90
	R^2^	0.61	0.51	0.58	0.55	0.24	0.43
ED_pca	RMSE	0.95	1.17	10.75	16.51	16.33	24.74
	R^2^	0.59	0.53	0.59	0.49	0.13	0.33
SAM_pca	RMSE	0.91	1.25	9.60	14.85	15.61	22.22
	R^2^	0.62	0.47	0.67	0.59	0.21	0.46

For pH, the performance sequence was as follows: SAM > SAM_pca > MD > ED_pca > ED (R^2^, 0.58–0.64; RMSE, 0.89–0.96); thus, SAM achieved the best accordance for pH. For SOC, the performance sequence was: ED > SAM > ED_pca > MD > SAM_pca (R^2^, 0.47–0.56; RMSE, 1.13–1.25%). Clearly different from the results for pH, ED and SAM yielded the best performance, both outperforming the similarity based on the reduced dimension of PC space (ED_pca and SAM_pca). It is important to note, important information may be lost if all PCs are not retained, even though the 15 PCs used explained almost all of the total observed variance of the spectra. The performance for CEC was: SAM_pca > ED > SAM > ED_pca > MD (R^2^, 0.58–0.67; RMSE, 9.60–10.83 cmol·kg^-1^). Thus, the samples selected by SAM_pca were the most similar (R^2^ = 0.67), while ED, SAM, ED_pca, and MD performed similarly (R^2^, 0.58–0.60).

For particle size distribution, percent clay content performance was generally superior to that of sand and silt. The performance sequence for clay was: SAM > SAM_pca > MD > ED > ED_pca (R^2^, 0.49–0.61; RMSE, 14.43–16.51%). The performance sequence for percent sand was: SAM > SAM_pca > MD > ED > ED_pca (R^2^, 0.33–0.55; RMSE, 20.33–24.74%), notably identical to clay. The accordance for percent silt was the lowest, and unique from sand and clay: SAM > ED > MD > SAM_pca > ED_pca (R^2^, 0.13–0.37; RMSE, 13.95–16.33%).

It is apparent that there is no single sequence of best indices per-property. The highest accordance (R^2^) achieved for CEC, pH, clay, SOC, sand, and silt were 0.67, 0.64, 0.61, 0.56, 0.55, and 0.37, respectively. No properties reached the level of good accordance (R^2^ ≥ 0.8), most fell within the range of moderate accordance (0.50 ≤ R^2^ < 0.80), and silt notably fell in the range of poor accordance (R^2^ < 0.5).

We hypothesized that the properties with strong, direct spectral responses, such as SOC, would have good accordance, while properties with low or indirect spectral responses, such as pH, would be poor; however, the accordance for SOC was only moderate. The reason may be that the spectral response of SOC is masked or disturbed by the presence of other soil properties, such as iron content [21]. The moderate performance of pH indicated that properties with no direct spectral response still had the potential to be well predicted through spectral pedo-transfer functions [22].

Predictably, when comparing similarity indices, different results were found. For example, the accordance for sand ranged from a moderate R^2^ = 0.55 (SAM), to a relatively low R^2^ = 0.33 (ED_pca). Thus, the selection of a proper similarity index is essential for determining suitable similar samples from spectral libraries of different scales. SAM provided the best or second-best performance for all six properties. SAM mainly considers the overall spectral shape, focusing less on the relative difference in reflectance. The performance of SAM_pca was better than SAM in some cases, but it performed poorly for sand (R^2^ = 0.46). ED and ED_pca values were very similar, with ED performing slightly better. Surprisingly, the differences between ED and MD were small as we expected MD to be superior since it accounts for covariance among bands, and the spectra were collected at high resolution and highly correlated.

### 3.3 Comparison between spectral and integrative compositional similarity

Pearson correlations between the spectral similarity evaluated by the five indices, and their corresponding integrative compositional similarity (represented by Euclidean distance of PCs converted from the six physiochemical properties) are presented in Table 3.

**Table 3:** The Pearson correlation coefficient between spectral and integrative compositional similarity.

Methods	SAM	ED	MD	ED_pca	SAM_pca
Correlation Coefficient	0.44	0.48	0.33	0.43	0.40

The correlation between integrative compositional and spectral similarity was significant (p < 0.01) for all five similarity indices, with ED being the highest (in contrast to its moderate performance in individual physiochemical property evaluation), and MD the lowest. The high correlation achieved by SAM was consistent with its good performance as evaluated by individual compositional similarity. The different trends in similarity index performance between compositional and individual compositional similarity indicates that the interactions between physiochemical properties and spectral responses are substantial; thus, an integrated measure is not simply the sum of the simple measures.

### 3.4 PLSR model prediction accuracy

As presented in Table 4, the high accordance between spectral similarity and SOC similarity was achieved by ED and SAM. For PLSR model prediction, similar samples selected by SAM yielded the best performance, followed by MD and ED. For pH prediction, SAM and ED achieved the highest predictive accuracy (RPD = 1.88), while the performances of SAM_pca and MD were only slightly less (RPD, 1.86–1.87). For CEC, model performances were inconsistent with other results, as the samples selected by SAM_pca were most similar in CEC content; however, the PLSR models based on similar samples chosen by SAM_pca performed relatively poorly (RPD = 1.49). Consistent with the highest accordance achieved by SAM and SAM_pca, the best PLSR models for clay were based on similar samples selected by ED_pca, SAM, and SAM_pca (RPD, 1.87–1.88). Interestingly, although the accordance for ED_pca was low, their PLSR models yielded strong performances. Consistent with the lowest accordance between spectral and physiochemical similarity, prediction accuracy for silt was the lowest compared to other properties (RPD, 1.18–1.26). The best models for silt were based on SAM and SAM_pca, which is also consistent with the above similarity analysis. For prediction of sand composition, the models with the highest accuracy were also based on SAM_pca and SAM (RPD, 1.49–1.53).

**Table 4:** Predictive accuracy (RPD) for physiochemical properties using PLSR models based on similar samples selected with different similarity indices.

Properties	ED	MD	SAM	ED_pca	SAM_pca
pH	1.88	1.86	1.88	1.83	1.87
SOC	1.51	1.52	1.67	1.45	1.49
CEC	1.58	1.52	1.53	1.55	1.49
clay	1.79	1.70	1.87	1.88	1.87
silt	1.24	1.20	1.26	1.18	1.26
sand	1.42	1.44	1.49	1.47	1.53

Aligning with the results between spectra and individual compositional similarity, the PLSR models built based on SAM or SAM_pca provided the best performance for all of the physiochemical properties, save for CEC. Compared to the other similarity indices, SAM and SAM_pca could select samples that were closer in composition similarity. There are two important variables influencing the prediction accuracy of the PLSR model in this study: sample size and the selected similarity index. When the sample size was fixed (250 in the present study), selecting more compositionally similar samples can achieve greater accuracy when using PLSR models for prediction. This aligns with our research hypothesis that the samples selected based on the optimal similarity index will achieve higher predictive accuracy for target samples. Considering the possible loss of information during PCA, the use of SAM is recommended over SAM_pca. The model based on ED was only slightly better than that of MD, in agreement with their low differences for similarity comparison.

The RPD values of the PLSR models fell within two ranges: (1) for silt prediction, RPD was < 1.4; and (2) for all other PLSR models and indices, RPD was between 1.4 and 2.0. No models achieved an RPD > 2.0, possibly because of the large variations and heterogeneity of the global soil spectral library. We evaluated the statistical difference (i.e., RPD) of the five similarity indices using a pairwise t-test. SAM was statistically superior to MD (p < 0.05), while SAM was also superior to the other three indices (ED, ED_pca, and SAM_pca), but their differences were not significant. Thus, MD is not recommended considering its relatively poor performance. Although the difference between SAM and SAM_pca was not statistically significant, the performance of SAM_pca was unstable (relatively poor predictive performances for SOC and CEC); thus further supporting the use of SAM over SAM_pca. As shown in Table 4, different similarity indices had a significant influence on the performance of the PLSR model for the physiochemical properties analyzed. For example, the RPD of SOC varied over a relatively wide range, from 1.45 to 1.67. Thus, the selection of a reliable similarity index is essential. The accordance information between spectral and compositional similarity can help select appropriate indices when one needs to select similar samples from a spectral library for the prediction of a target sample. In addition, as revealed by the results of the similarity analysis and PLSR models, the properties that have high accordance with individual composition and spectral similarity, for example, pH and clay in this study, can be more accurately predicted using PLSR models. In contrast, the accordance for silt and sand was low; therefore, their PLSR models performed poorly. Thus, the relationship between individual composition and spectral similarity can be used as an indicator of the potential of VNIR spectroscopy for the prediction of different properties.

## 4. Conclusions

Compared to other similarity indices, SAM and SAM_pca selected samples were more compositionally similar to the target samples. Based on the similar samples selected by these indices, PLSR models achieved the highest predictive accuracy for all six of the soil physiochemical properties analyzed, except CEC. SAM is recommended over SAM_pca considering the possible loss of information during PCA analysis. The findings support the hypothesis that the accordance information between spectral and compositional similarity can help select appropriate indices when one needs to select similar samples from a spectral library for predicting target samples.

## Supporting information

S1 Fig (TIF)
