# Supplementary figures and images for: How similar is “similar,” or what is the best measure of soil spectral and physiochemical similarity?
Source: PLoS One. 2021 Mar 25;16(3):e0247028. doi: 10.1371/journal.pone.0247028 (PMC7993829; doi:10.1371/journal.pone.0247028)

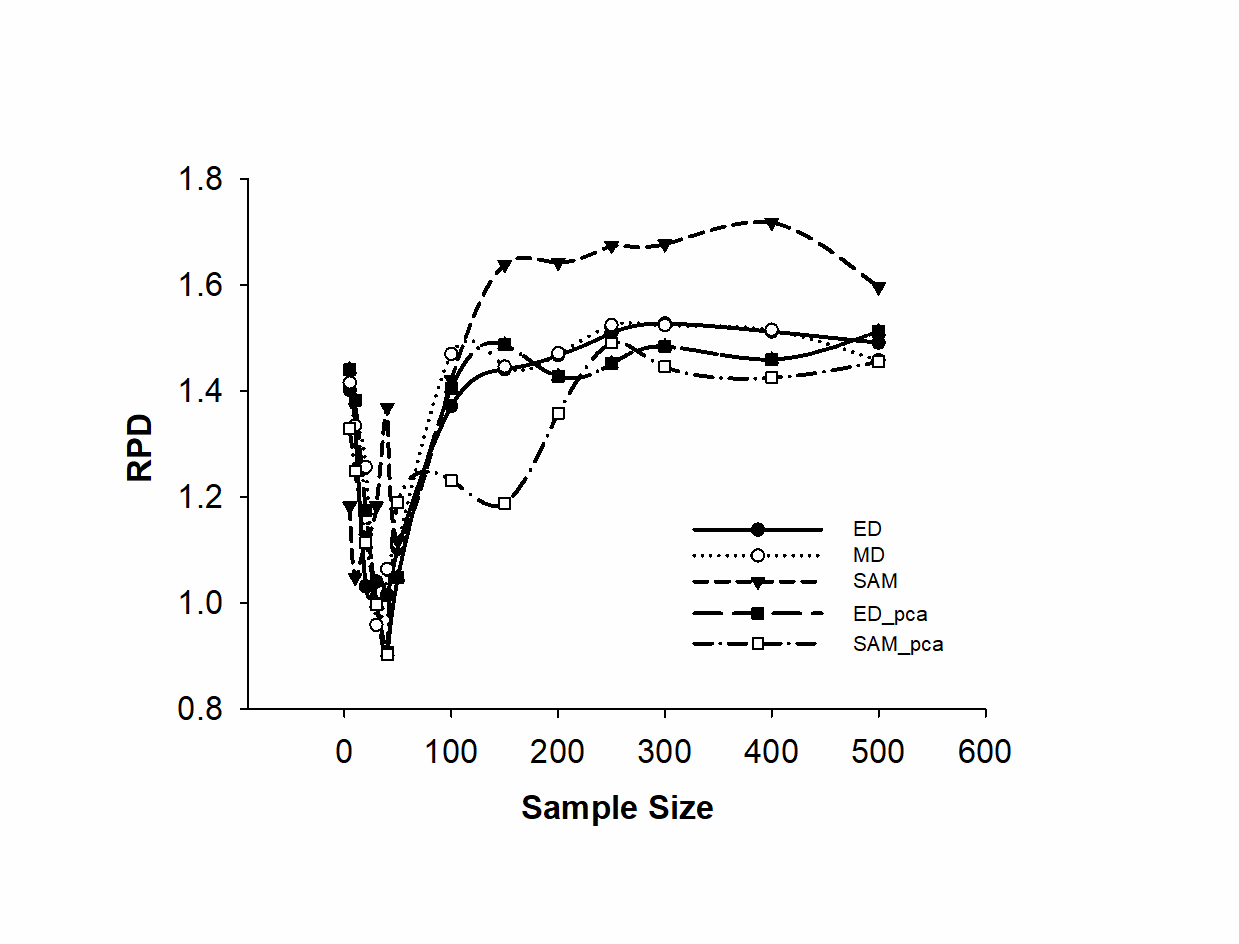

Supplement: S1 Fig — (TIF) [file pone.0247028.s001.tif]
